# Centrosomal abnormalities, multipolar mitoses, and chromosomal instability in head and neck tumours with dysfunctional telomeres

**DOI:** 10.1038/sj.bjc.6600438

**Published:** 2002-07-02

**Authors:** D Gisselsson, T Jonson, C Yu, C Martins, N Mandahl, J Wiegant, Y Jin, F Mertens, C Jin

**Affiliations:** Department of Clinical Genetics, University Hospital, SE-221 85 Lund, Sweden; Laboratory of Cytometry and Cytogenetics, Portuguese Institute of Oncology, Lisboa, Portugal; Laboratory of Cytochemistry and Cytometry, Department of Molecular Cell Biology, Leiden University Medical Center, Leiden, the Netherlands

**Keywords:** squamous cell carcinoma, pleomorphic adenoma, telomere, breakage-fusion-bridge cycle, centrosome

## Abstract

Carcinomas of the head and neck typically exhibit complex chromosome aberrations but the underlying mutational mechanisms remain obscure. Evaluation of cell division dynamics in low-passage cell lines from three benign and five malignant head and neck tumours revealed a strong positive correlation between multipolarity of the mitotic spindle and the formation of bridges at anaphase in both benign and malignant tumours. Cells exhibiting a high rate of mitotic abnormalities also showed several chromosome termini lacking TTAGGG repeats and a high frequency of dicentric chromosomes. Multicolour karyotyping demonstrated a preferential involvement in structural rearrangements of chromosomes with deficient telomeres. The majority of malignant, mitotically unstable tumours expressed the reverse transcriptase subunit of telomerase. These data indicate that some of the genomic instability in head and neck tumours is initiated by telomere dysfunction, leading to the formation of dicentric chromosomes. These form chromosome bridges at mitosis that could prevent the normal anaphase-telophase transition. In turn, this may cause an accumulation of centrosomes and mitotic multipolarity. Telomerase expression does not confer total stability to the tumour genome but could be crucial for moderating the rate of chromosomal evolution.

*British Journal of Cancer* (2002) **37**, 202–207. doi:10.1038/sj.bjc.6600438 www.bjcancer.com

© 2002 Cancer Research UK

## 

Carcinoma of the head and neck is the sixth most common malignancy world-wide (Parkin *et al*, 1988). Whereas tumours confined to the oral cavity and the airways are predominantly squamous cell carcinomas, tumours of the salivary glands are histologically more heterogeneous, with the most common type being pleomorphic adenoma (PA). Head and neck squamous cell carcinomas (HNSCC) are characterised by complex, non-random chromosomal changes. Common structural rearrangements include isochromosomes for 1q, 3q, 5p, and 8q, deletions of 3p, and homogeneously staining regions containing amplified material from 11q13 (Van Dyke *et al*, 1994; Jin *et al*, 1995). Losses and gains of whole chromosomes are also common, including -Y, +7, −17, −18, +20, and −21. Salivary gland PA, on the other hand, typically display simple structural changes of 8q11-12 or 12q13-15, leading to dysregulated expression of the *PLAG1* and *HMGA2* (formerly *HMGIC*) genes, respectively (Geurts *et al*, 1997; Kas *et al*, 1997). Rearrangements of *PLAG1* have also been detected in malignant salivary gland tumours, carcinoma ex pleomorphic adenomas, usually together with additional cytogenetic changes (Jin *et al*, 2001).

The mechanisms behind chromosome aberrations in head and neck tumours are obscure. Mitotic instability including breakage and/or nondisjunction of chromosomes has been demonstrated in established cell lines from HNSCC (Saunders *et al*, 2000). Recently, it has been suggested that this type of chromosome instability could be triggered by telomere dysfunction, leading to instability of chromosome termini (Artandi *et al*, 2000; Gisselsson *et al*, 2001b). Another mode of mitotic instability found in HNSCC cell lines consists of multipolar cell divisions, possibly caused by supernumerary centrosomes (Saunders *et al*, 2000). Little is known about the relation between centrosome abnormalities and mitotic multipolarity, on the one hand, and telomeric dysfunction and mitotic chromosome breakage on the other. However, the fact that structural and numerical chromosome abnormalities occur together in many tumours indicates that these processes may be related mechanistically. This study is an attempt to elucidate some of the events leading to complex karyotypic abnormalities in head and neck tumours by parallel evaluation of cell division figures, centrosomal configuration, telomere status, and telomerase expression in low-passage cell lines from HNSCC and PA.

## MATERIALS AND METHODS

### Cell culture

Biopsies from five HNSCC and three PA (Table 1

**Table 1:**
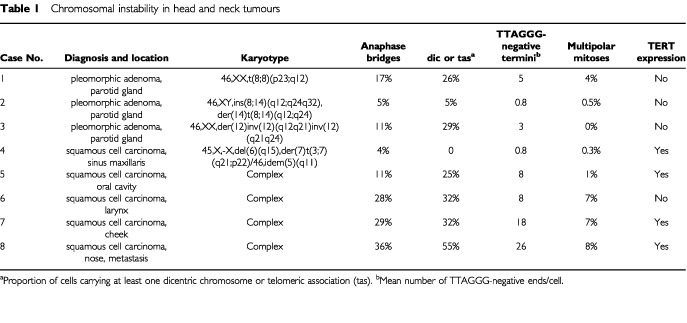
Chromosomal instability in head and neck tumours

) were minced with scissors and disaggregated overnight in 180 U ml^−1^ collagenase II (Cooper Biomedical, Lakewood, NJ, USA). The resulting suspension was plated on vitrogen-coated chamber slides or in culture flasks. Cells were cultured in keratinocyte serum- free medium (GIBCO 041 – 17005 M), supplemented with 0.23 mg ml^−1^ L-glutamine, 100 IU ml^−1^ penicillin, 0.2 mg ml^−1^ streptomycin, and 2.5 μg ml^−1^ amphotericin. Cell culture morphology was assessed in an inverted microscope. After partial harvesting and a first chromosome banding analysis, the remaining cells were further cultured until 90% confluence (3–5 days) and then subcultured 3–5 times before analysis, except case five, which was subcultured 15 times. As a negative control, dermal fibroblasts subcultured four times were used.

### Analysis of mitotic cell morphology

For analysis of mitotic figures, cells on chamber slides were washed in phosphate-buffered saline (PBS) for 5 min, fixed in methanol : acetic acid (3 : 1) at −20°C for 30 min, air-dried, and stained with haematoxylin and eosin. At least 150 anaphase and 150 metaphase cells were analysed in each case.

### Centrosome detection

Cells on chamber slides were washed in PBS for 5 min, fixed in methanol at −20°C for 30 min and air-dried. The cells were then rehydrated in PBS for 5 min, blocked with 1% bovine serum albumin in PBS for 15 min, and incubated with murine monoclonal anti-γ-tubulin antibodies (GTU-88, Sigma, St. Louis, MS, USA), diluted 1 : 40 in blocking buffer for 30 min. After washing three times for 3 min in PBS, secondary 1 : 20 biotinylated anti-mouse antibodies (E0354, DAKO A/S, Denmark) were applied for 30 min, followed by washing in PBS three times for 3 min, and incubation with 1 : 50 streptavidin-Alexa 594 (Molecular Probes, Leiden, the Netherlands). β-tubulin was detected subsequently, using 1 : 40 dilutions of mouse monoclonal anti-β-tubulin antibodies (2-28-33, Sigma) followed by anti-mouse antibodies coupled to fluorescein-isothiocyanate (F0232, DAKO A/S). Cells were dehydrated in a 75-85-100% ethanol series and counterstained with 4′,6-diamidine-2′-phenylindole (DAPI). At least 50 cells were evaluated in each case.

### Metaphase chromosome analysis

Cultures were harvested by exposure to 0.01 μg ml^−1^ colchicine for 12–16 h, followed by hypotonic treatment in 0.06 M KCl for 35 min, and fixation by 3 : 1 methanol : acetic acid. Chromosome banding was with Wright's stain according to standard methods (Mandahl, 2001). Multicolour karyotyping was performed by the COBRA probe set as previously described (Jin *et al*, 2001). Telomeric TTAGGG repeats were visualised by fluorescence *in situ* hybridisation (FISH) with fluorescein-conjugated (CCCTAA)_3_ peptide nucleic acid probes (Landsdorp *et al*, 1996). Signal intensity was directly quantified by the Cytovision software (Applied Imaging, Newcastle, UK). Chromosome identification was by computerised inversion and enhancement of DAPI bands. Centromeric regions were detected using the human pan-alpha satellite probe (Cambio, Cambridge, UK). The *PLAG1* locus was analysed by PAC clones 233, 234, and 235, kindly provided by Dr J Bullerdiek, Bremen, Germany. At least 20 cells were evaluated in each case.

### Detection of the telomerase regulatory subunit

Cultures parallel to those used for chromosome and cell division analyses were subjected to total RNA extraction and cDNA synthesis according to standard procedures. The reverse transcriptase subunit of telomerase (*TERT*) was coamplified with an *ACTB* fragment in a PTC-225 temperature cycler (MJ Research, Cambridge, MA, USA). An initial 5-min step at 96°C was followed by 35 cycles at 96°C for 30 s, 60°C for 30 s, and 72°C for 1.5 min, and a final extension at 72°C for 10 min. Primers for *ACTB* (Raff *et al*, 1997) were added after 10 cycles. Amplified products were separated on a 1.8% agarose gel and visualized by Vista Green nucleic acid gel stain (Amersham Pharmacia, Amersham Place, UK). A 145-bp segment outside the alternatively spliced region was amplified by using the primers TERT F1784 (CGGAAGAGTGTCTGGAGCAA) and TERT R1928 (GGATGAAGCGGAGTCTGGA), and alternative splice products of the *TERT* transcript were amplified by using the primers TERT F2164 (GCCTGAGCTGTACTTTGTCAA) and TERT R2620 (CGCAAACAGCTTGTTCTCCATGTC) as described (Ulaner *et al*, 1998).

## RESULTS

### Mitotic and centrosomal abnormalities

**Figure 1:**
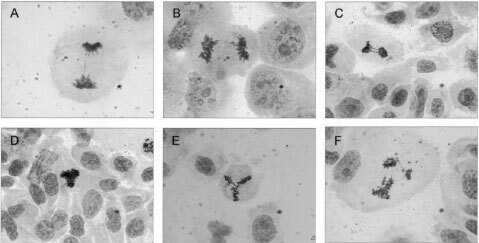
Broken (**A**) and intact (**B** and **C**) anaphase bridges in cases 2, 5 and 8, respectively, and multipolar metaphase (**D** and **E**) and anaphase (**F**) cells in case 8; haematoxylin-eosin staining.

**Figure 2:**
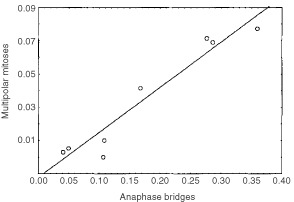
Positive correlation *r*=0.96 (Pearson; *P*<0.05) between the frequency of multipolar mitoses and anaphase bridges.

**Figure 3:**
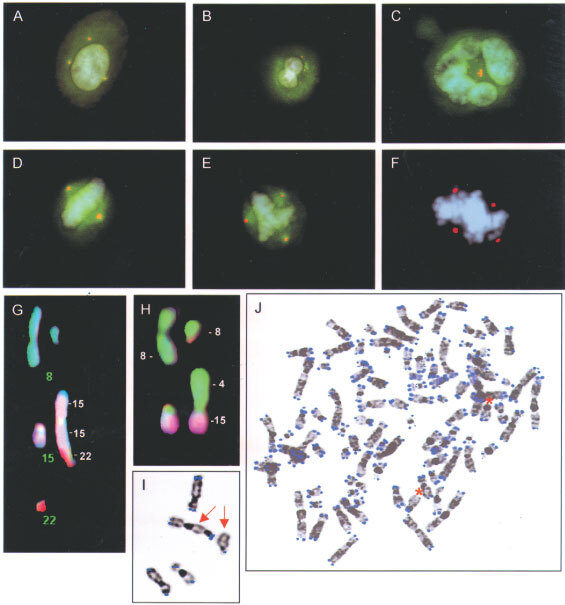
Multiple centrosomes (γ-tubulin, red) in mononucleated (**A**), binucleated (**B**), and pentanucleated cells (**C**) in cases 1, 2, and 8, respectively. Normal (**D**) and multipolar (**E** and **F**) mitotic figures in cases 5, 8, and 4, respectively; the β-tubulin fluor layer (green) has been removed for clarity in (**F**). Non-clonal telomeric associations between two chromosomes 15 and one chromosome 22 (**G**) and an unbalanced 4;15-translocation (**H**) in addition to the clonal 8;8-translocation (**G** and **H**, top row) demonstrated by multicolour karyotyping in case 1. Another cell from the same case shows absence of TTAGGG signals at the q termini of two chromosomes 15, one of which is involved in a telomeric association (**I**). In other cells, absence of TTAGGG signals was observed at several termini (**J**), including 15p and q (asterisks).

Anaphase bridges were found by haematoxylin-eosin staining in all eight cell lines (Table 1; Figure 1A–C

). The four tumours containing the highest number of anaphase bridges (cases 1, 6–8) also exhibited tri-, tetra-, and hexapolar mitotic figures at frequencies of 4%-8% (Figure 1D–F). There was a strong positive correlation (Pearson, *r*=0.96, *P*<0.05; Figure 2

) between the frequencies of bridges and multipolar mitoses. No bridges or multipolar mitoses were found in control cultures of dermal fibroblasts. Immunofluorescence showed one or two centrosomes in the majority of cells in all tumour cases, similar to the pattern seen in fibroblasts. All cell lines also contained a population of cells with large (approximately double-sized) nuclei, showing larger centrosomes, typically with 3–8 centrioles assembled in one cluster. Such clusters were also observed in the fibroblasts, although never containing more than four centrioles per cluster. The cases with the highest rate of bridges and multipolar divisions (cases 1, 6–8) also showed a wide variety of centrosomal configurations, including multiple, scattered or clustered centrosomes at interphase, as well as metaphase cells with several microtubule organising centres (Figure 3A–F

). None of these patterns were observed in the other cell lines or fibroblast cultures. Approximately half of the interphase cells with centrosomal abnormalities were multinucleated.

### Metaphase chromosome analysis

Clonal chromosome abnormalities were present in all tumours (Table 1). Two of the adenomas (cases 1 and 2) contained unbalanced rearrangements of chromosome 8. FISH analysis with probes covering *PLAG1* in 8q12 showed split signals in both cases (data not shown). The third case of adenoma exhibited a double inversion with breaks in 12q12, q15, and q32. Previous analyses had shown that the 12q15 breakpoint was distal to the *HMGA2* gene (Gisselsson *et al*, 2000). One of the squamous cell carcinomas (case 4) showed rearrangements only of chromosomes 3, 5, 6, and 7. The four other carcinomas had complex karyotypes (>five changes), including homogeneously staining regions in cases 6–8. Additional, non-clonal abnormalities were found in >5% of cells in cases 1, 3 and 5–8, but in <1% of cells in cases 2 and 4. Pan-alpha satellite detection revealed a low frequency (<5%) of dicentric chromosomes (dic) or telomeric associations (tas) in the latter two tumours, whereas the other cases showed dic or tas in >25% of cells. FISH detection of TTAGGG repeats showed a mean of 0.8 ends without signals in cases 2 and 4, similar to the rate in fibroblasts (0.5). The other cases showed between 3 and 26 TTAGGG-negative chromosome ends per cell. The frequency of anaphase bridges was positively correlated to both the mean number of TTAGGG-negative termini (*r*=0.90; *P*<0.05) and the frequency of cells with dic/tas (*r*=0.88; *P*<0.05).

Since case 1 was a benign tumour with a single clonal abnormality but nonetheless showed a very high rate of anaphase bridges and multipolar mitoses, it was selected for a more detailed analysis. G-banding of cells from passage 3 revealed non-clonal abnormalities in 11/30 cells analysed, the majority of which were telomeric associations. In the primary culture, G-banding had shown non-clonal abnormalities in 18/100 cells. Multicolour karyotyping of cells from passage 3 corroborated these results, showing abnormalities in addition to the t(8;8) in 13/30 cells (Figure 3G,H), approximately half of which were telomeric associations. Other aberrations were unbalanced translocations (five cells) and dicentric chromosomes (two cells). Chromosome 15 was most commonly affected by telomeric associations and structural rearrangements, involving 15p in one cell, 15q in four cells, and both arms in five cells. Chromosome 19 was the second most commonly involved, being rearranged in three cells. FISH detection of telomere repeat sequences was combined with computerised inversion of DAPI banding in order to measure the distribution of negative ends among chromosomes (Figure 2I,J). The most common negative terminus was 15q (16/30 cells), followed by 15p (15/30), 9p (7/30), 19q (6/30), 16p (6/30), 21q (5/30) 9q (4/30), and 19p (4/30).

### Expression of the telomerase reverse transcriptase subunit

**Figure 4:**
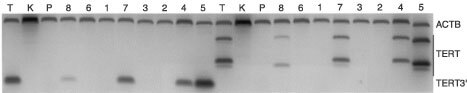
TERT RNA expression measured by reverse transcriptase–PCR with primers outside the alternatively spliced region (TERT3′, left) and primers for four alternative splice products (TERT, right). TERT3′ expression as well as two major (239 and 421 bp) and two minor (275 and 457 bp) TERT products are present in thymus (T) and the HNSCC cases 4, 5, 7 and 8, but not in kidney (K), pancreas (P), or the other head and neck tumours; β-actin (ACTB) was used as internal control.

Human telomerase activity is determined by the expression of its reverse transcriptase subunit, TERT. However, the *TERT* transcript is alternatively spliced, and only the expression of full-length messages correlates with enzyme activity (Ulaner *et al*, 1998). Reverse transcriptase–PCR analysis revealed expression of full-length TERT in all HNC except in case 6, but in none of the PA (Figure 4

). Full-length TERT was also found in thymus, used as a positive control, but not in normal kidney and pancreas, used as negative controls.

## DISCUSSION

An inherent genetic instability in neoplastic cells allows rapid accumulation of mutations favourable to tumour progression. Though several different modes of chromosomal instability have been reported, the underlying molecular mechanisms are poorly known (Lengauer *et al*, 1998). Multipolar mitoses of various configurations may be caused by an abnormal structure and/or function of the mitotic spindle machinery (Lingle *et al*, 1998; Lingle and Salisbury, 1999; Ghadimi *et al*, 2000; Shono *et al*, 2001). In particular, centrosomal aberrations have been correlated with a number of different genetic abnormalities in tumours, e.g. amplification of *STK15* (Zhou *et al*, 1998), mutations in *TP53* (Carroll *et al*, 1999), and inactivation of *BRCA1* (Xu *et al*, 1999), *BRCA2* (Tutt *et al*, 1999), and *GADD45* (Hollander *et al*, 1999). In adenocarcinomas of the prostate and breast, centrosomal abnormalities are known to increase in parallel to loss of tissue differentiation and the development of aneuploidy (Pihan *et al*, 2001; Lingle *et al*, 2002). Furthermore, abnormal centrosome function can be induced by expression of the papilloma virus genes E6 and E7, inhibiting normal TP53 and RB1 activity, respectively (Duensing *et al*, 2000).

Another mode of mitotic instability consists of the formation of chromosome bridges at anaphase. This phenomenon has recently been described in several tumour types, including pancreatic and ovarian adenocarcinoma, HNSCC (Gisselsson *et al*, 2000; Saunders *et al*, 2000), osteosarcoma, and soft tissue sarcoma (Gisselsson *et al*, 1999). Such bridges may consist of mitotically unstable chromosomes undergoing repeated breakage-fusion-bridge (BFB) cycles, thus causing massive reorganisation of chromosome structure (McClintock, 1940). However, chromosome banding data have demonstrated that many tumours exhibiting complex structural rearrangements also show a high number of gains and losses of whole chromosomes, suggesting a mechanistic link between structural chromosome instability and the generation of aneuploidy (Mitelman Database of Chromosome Aberration in Cancer, 2002).

In this study, a strong correlation (*r*=0.96) was found between the frequencies of anaphase bridges and multipolar mitoses in head and neck tumours. One explanation for this could be that both these phenomena depend on one, so far unknown, genetic or epigenetic abnormality. For instance, dysregulation of mitotic control mechanisms could allow both multipolar cell divisions and anaphase bridging with little negative effect on cellular survival (Li and Nicklas, 1995). Alternatively, anaphase bridging and mitotic multipolarity may be correlated because one of the phenomena is directly dependent on the other. Previous investigations have demonstrated that anaphase bridges need not always break at the anaphase-telophase transition; instead they may remain as strings of chromatin between interphase nuclei (Gisselsson *et al*, 2001a). These internuclear connections could, in turn, prevent cytokinesis and lead to duplication of both chromosome and centrosome number. Depending on the extent to which centrosomal duplication and separation occur in the subsequent cell cycle, the next division could then be orchestrated by two, three, or four centrosomes (Boveri, 1914). In our material, we observed multipolar mitoses clearly containing an increased amount of chromosome material (Figure 3F). However, it seems implausable that the cells with a very high number of nuclei (Figure 3C) could undergo further functional cell divisions; rather they may represent an evolutionary dead end. This is in accordance with the observation that mitoses with five or more poles were very rare (<0.3%).

Recent data from aggressive tumours with a high rate of chromosomal instability, such as pancreatic adenocarcinoma and osteosarcoma, have shown that BFB cycles can be triggered by telomere shortening (Gisselsson *et al*, 2001b). In this study, the frequency of anaphase bridges in HNSCC and PA was, in fact, positively correlated with the number of chromosome ends without TTAGGG signals, indicating that telomere shortening may cause chromosomal instability also in these neoplasms. Furthermore, a detailed multicolour analysis of case 1 demonstrated that the chromosome most commonly lacking termimal TTAGGG repeats was also the one involved in most of the non-clonal aberrations. These chromosome changes were not limited to telomeric associations, but also included dicentrics and other, more complex unbalanced rearrangements. Similar aberrations have been observed in tumours from transgenic mice with dysfunctional telomeres (Artandi *et al*, 2000).

The present study thus indicates that a single process could lead both to structural and numerical chromosome instability. Telomere shortening will trigger BFB events that, in turn, may prevent normal cytokinesis and thereby lead to accumulation of centrosomes and multipolar cell divisions. This scenario does not appear to be limited to malignant cells. Cases 1 and 3 were both classified as benign PA, but exhibited a high frequency of anaphase bridges. Investigations of colorectal polyps in mice have shown that BFB events may actually occur already at the dysplastic stage (Rudolph *et al*, 2001). In man, mitotically unstable ring chromosomes, telomeric associations and anaphase bridges are common in borderline malignant mesenchymal tumours, including giant cell tumours of bone and atypical lipomatous tumours (Mandahl *et al*, 1998; Gisselsson *et al*, 1999). However, this is the first study demonstrating all these phenomena in benign lesions. It is noteworthy that cytogenetic alterations similar to those found in case 1, albeit clonal, are typically present in carcinomas arising from pleomorphic adenomas (Jin *et al*, 2001). The triggering of mitotic multipolarity by BFB instability may thus be one important mechanism for the development of complex karyotypes and malignant transformation in head and neck tumours.

Full length telomerase reverse transcriptase was expressed in four of the five HNSCCs but in none of the PAs. This is in accordance with earlier studies, showing expression of telomerase in approximately 90% of malignant tumours (Artandi and DePinho, 2000). The precise role of telomerase in carcinogenesis is not known. It has been suggested that this enzyme is essential for stabilising the genome of malignant tumours. However, in this study there was no difference in the frequency of BFB events between the tumours expressing TERT and those that did not. These results indicate that the stabilising effect of telomerase expression on the tumour genom is incomplete. It should be noted that that stabilisation of chromosome breaks could also be achieved through telomere capture (Slijepcevic and Bryant, 1998; Gisselsson *et al*, 1999). This does not rule out that TERT expression plays a vital role in tumorigenesis. Clonal expansion in the absence of telomere lengthening implicates extensive erosion of TTAGGG repeats at all chromosome termini. The resulting global genomic instability would probably be incompatible with cellular survival. Even a partial telomere-lengthening effect could then be beneficial by keeping genomic instability at a moderate rate, still allowing evolution of clonal chromosomal imbalances favourable to tumour progression. In this context, drugs inhibiting telomerase activity could be highly effective, potentially tilting the balance from a moderate rate of cytogenetic evolution towards complete mitotic failure.
